# Substitute for Wine

**Published:** 1863-10

**Authors:** Wm. A. Hammond

**Affiliations:** Surgeon-General; Surgeon-General’s Office, Washington


### SUBSTITUTE FOR WINE.

SURGEON-GENERAL'S OFFICE,
*Washington*, August 15, 1863.

*Circular No.* 13.—Surgeons-in-charge of hospitals are informed that they can procure a substitute for *Port Wine*, for the use of the sick, by making proper requisition therefor.

In consequence of the impossibility of procuring pure port wine of the grade formerly issued to the army, an article of Tanagona wine has been adopted for issue instead.

This wine is light, dry, and astringent, and is the pure juice of the grape, purchased by the Medical Department in bond, and bottled at medical purveying establishments.

WM. A. HAMMOND,
Surgeon-General.

